# Cortical Blindness due to Bilateral Occipital Infarcts in a Renal Failure Patient with Prostate Cancer: A Rare Complication of Hemodialysis

**DOI:** 10.1155/2013/539761

**Published:** 2013-01-09

**Authors:** O. G. Doluoglu, M. S. Saricaoglu, C. V. Oztekin, A. Karakurt, A. O. Akdemir, E. Koc

**Affiliations:** ^1^Department of Urology II Clinic of Ankara Numune Education and Research Hospital, 06100 Ankara, Turkey; ^2^Department of Ophthalmology III Clinic of Ankara Numune Education and Research Hospital, 06100 Ankara, Turkey; ^3^Department of Nephrology Clinic of Ankara Numune Education and Research Hospital, 06100 Ankara, Turkey

## Abstract

Loss of vision is a rare complication seen in hemodialysis patients. It is thought to develop because of the hypotension that can be observed during dialysis. This paper involves a patient with acute loss of vision during hemodialysis due to bilateral occipital infarcts.

## 1. Introduction

Acute complications commonly occur during routine hemodialysis treatments. They include the following [1]: hypotension (25–55%), cramps (5–20%), nausea and vomiting (5–15%), headache (5%), chest pain (2–5%), back pain (2–5%), itching (5%), and fever and chills (<1%).

Acute loss of vision can be observed in hemodialysis patients. It may be seen due to ischemic optic neuropathy (ION) or cerebral cortical infarcts caused by hypotension during the process. Cortical blindness is a very rare complication associated with hemodialysis, which to our best knowledge, is defined in only two reports in the literature [2, 3]. This paper presents a patient with acute, bilateral, and complete loss of vision observed during hemodialysis, proved to be central and due to ischemic infarcts in the bilateral occipital lobes. 

## 2. Case Report

A 65-year-old male patient presented to our institution with lower urinary tract symptoms and elevated serum urea and creatinine levels. He was under followup for type II diabetes mellitus for the last 6 years and prostate cancer for the last 3 years. Maximal androgen deprivation therapy (GnRh analogue and nonsteroidal antiandrogen) was initiated 3 years ago, but the patient did not receive the treatment in a regular way. He had had 2 sessions of hemodialysis previously in emergency settings in another center. Blood tests at the time of first inspection were serum urea: 135 mg/dL, creatinine: 6.2 mg/dL, and prostate specific antigen: >153 ng/dL. Evaluation of the urinary tract revealed a maximal urinary flow rate of 6 mL/s, an international prostate symptom score (IPSS) of 29, and grade III dilation of bilateral kidneys and ureters on the sonogram. Invasion of the trigone and left ureteral orifice was observed, and right unit was double-j stented during cystoscopy. Diversion of the left unit with a percutanous nephrostomy was recommended, but was refused by the patient. Transurethral resection of the prostate (TUR-P) was performed after serum creatinine was stabilized at a level of approximately 3 mg/dL 1 month after stenting of the right collecting system. Patient was discharged with a serum creatinine level of 2.9 mg/dL and with conservative recommendations like increased oral hydration and low-protein diet. The patient was meticulously followed after the TUR-P with monthly routine blood biochemistry and PSA measurements and urinary ultrasound. Four months later, the patient applied with a sudden onset tachycardia. Laboratory analysis revealed serum hemoglobin, total calcium, ionized calcium, urea, creatinine, potassium, and glucose levels of 8.7 g/dL, 8.2 mg/dL, 4.7 mg/dL, 87 mg/dL, 3.61 mg/dL, 7.2 mmol/L, and 190 mg/dL, respectively. Coagulation panel and urine analysis were normal. There were wide QT interval and upright T waves in the Electocardiogram (ECG). Nephrologist suggested infusion of Ca and initiation of hemodialysis by these findings. The baseline and predialysis blood pressures of the patients were 147/85 mmHg and 150/90 mmHg, respectively. 

A dialysis duration of 2.5 hours was scheduled in order to achieve the target uro-reduction rate of 30%. 1.2 m^2^ synthetic biocompatible membrane (Fresenius F6 polisulfone low flux fully synthetic biocompatible membrane) was employed. Dialysate bicarbonate value was set to 30 mEq/L. Heparin was not used as this was the first hemodialysis of the patient.

During dialysis, acute bilateral loss of vision developed in the patient and therefore the intervention was terminated. The highest and lowest systolic/diastolic blood pressures recorded during hemodialysis were 90/60 and 80/50 mmHg, respectively. 

**Figure 1:**
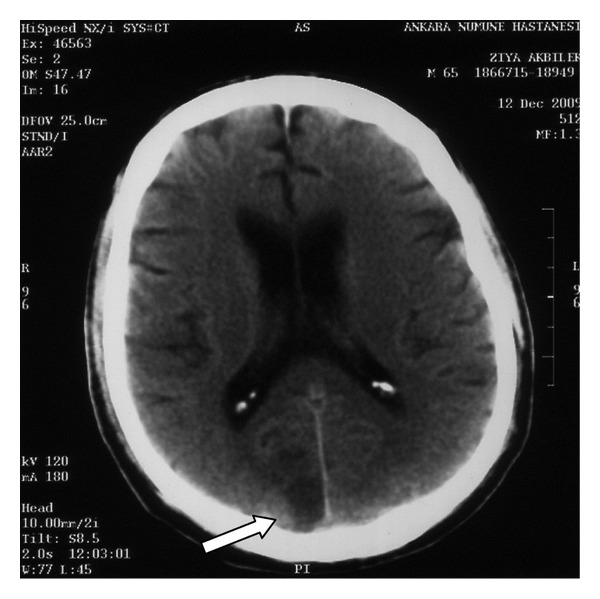
Right occipital infarct area in the CT scan (white arrow).

**Figure 2:**
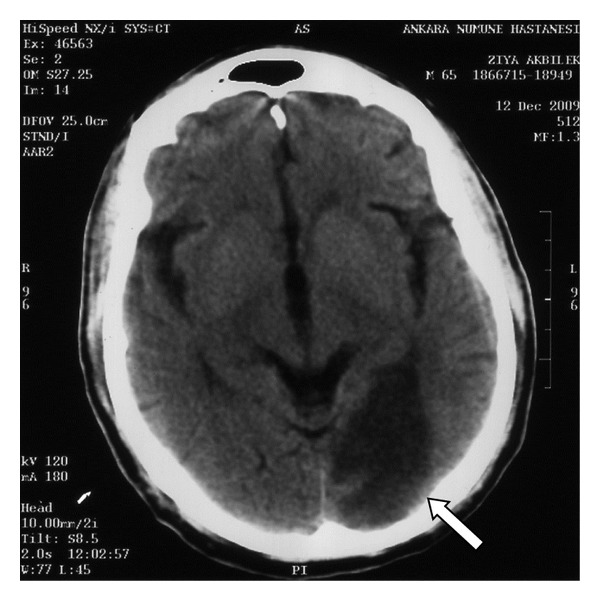
Left occipital infarct area in the CT scan (white arrow).

Ophthalmological inspection revealed that patient's vision was at the hand-motion level, the light reflexes were normal, there was no afferent pupillar defect, bilateral posterior segments, and bilateral intraocular pressures were normal. Findings suggested a central pathology, and the CT scan performed afterwards revealed large hypodense infarct areas in the bilateral occipital and parasagittal posterior region of right parietal lobes (Figures 1 and 2). Bilateral carotid artery doppler and echocardiography for differential diagnosis of thromboembolism were normal.

Patient was conscious, cooperating, and well oriented, and all deep tendon reflexes were found to be hyperactive in the neurological evaluation. Also a few days later right hemiparesis appeared in the patient. Therefore patient was accepted in the intensive care unit of the neurology department and received two times of 0,6 cc clexane and three times 10 mg dexametasone a day. Thus our purpose was to protect the ischemic penumbra with anticoagulant and anti-inflammatory effect. 

Despite the recovery of hemiparesis, loss of vision continued during followup of the patient. Patient's loss of vision continued and did not recover throughout the followup.

## 3. Discussion

Renal Failure (RF) is loss of renal function best diagnosed and characterized with increased serum creatinine levels. It can be categorized into two major groups as acute and chronic RF. Acute RF can be further subgrouped into prerenal, intrinsic (renal), or postrenal RF's. Furthermore, acute RF can arise during the course of a chronic RF in 13% of the patients [4].

We hereby present a patient with RF due to severe bladder outlet obstruction together with the invasion of the trigone and obstruction of ureteral orifices, caused by prostate cancer. It was reported in a study that invasion of prostate cancer was observed in lymph nodes, bones, lungs, and bladder, liver and adrenal in decreasing order in autopsy series [5]. 

Once RF develops, either acute or chronic, hemodialysis is always a treatment option on the table. Indications of hemodialysis include volume overload, severe hypercalcemia, severe metabolic acidosis, pericarditis, and other specific symptoms associated with azotemia [6, 7].

Loss of vision is a rare complication of hemodialysis which is thought to be associated with hypotension observed during the process [8]. On the other hand, hypotension is the most common complication of hemodialysis which is still observed in 20–30% of the patients despite the advances in medical technologies [9].

Loss of vision associated with hemodialysis may develop as a result of ION or cerebral cortical infarcts [10, 11]. ION can either be anterior or posterior. Anterior ION may present with edema of the optic nerve and peripapillary hemorrhage. In the cases of posterior ION however, optic disc is always normal on examination [8, 12]. Likewise in our case, pupillary reactions were positive and there was no afferent pupillary defect, and bilateral fundi were normal. These findings suggested a central pathology, most likely a cortical loss of vision due to occipital infarcts, which was later confirmed by the neuroradiological imaging.

The visual cortex is supplied by the posterior cerebral artery, and mainly its calcarinal branch. Also there is a rich anastomosis in the posterior pole, formed by the terminal branches of the posterior and middle cerebral arteries which is specifically important for the macular visual cortex. Vascular obliterations may cause loss of vision and impair visual field, generalized hypoperfusion of the cortex may result in severe loss of vision as is the case in our patient. Natural history of cortical blindness is unpredictable and yet to be fully understood as some may resolve spontaneously while others may not. The loss of vision in our patient was accepted to be irreversible as no improvements were observed in vision over time and his clinical status remained stable throughout the followup. 

In order to prevent the visual complications during hemodialysis, changes in blood pressures of the patients during the process should be thoroughly monitored together with their general uremic and biochemical status; and any problem arising should be handled in a multidisciplinary approach including departments of urology, nephrology, and ophthalmology.
